# CHALLENGES FACED BY OLDER PERSONS IN USING PRESCRIPTION MEDICATION LABELS: WHAT NEEDS TO CHANGE?

**DOI:** 10.1093/geroni/igz038.2606

**Published:** 2019-11-08

**Authors:** Rahul Malhotra, Sumithra Suppiah, Yi Wen Tan

**Affiliations:** Duke-NUS Medical School, Singapore, Singapore

## Abstract

In Singapore, while many older people cannot read English, prescription medication labels (PMLs) are predominantly dispensed in English. This qualitative study documented the challenges faced and solutions employed by users (i.e. older Singaporeans) and dispensers (i.e. pharmacy staff) of PMLs. In total, 30 in-depth interviews were conducted; 20 were equally divided between older Singaporeans (≥60 years) who could read English and those with limited/no English reading ability, and 10 were conducted with pharmacy staff across 6 polyclinics. The audio-taped interviews were transcribed verbatim and analysed thematically. The interviews with older Singaporeans and pharmacy staff revealed similar challenges in using PMLs. The first challenge related to reading and understanding PMLs by older people, mainly due to their limited English proficiency (LEP) or illiteracy. Consequently, older Singaporeans often relied on family members, domestic workers or pharmacy staff to help them interpret PMLs. Specifically, to address LEP, pharmacy staff reported translating PML instructions verbally and also handwriting them on PMLs. For illiterate patients, pharmacy staff reported drawing illustrations on PMLs to communicate key medication information. The second challenge related to PML readability, due to small font size. To address this, pharmacy staff routinely re-wrote medication information on PMLs in larger handwriting. Such improvised solutions by pharmacy staff to address the challenges faced by older Singaporeans in using PMLs indicate a pressing need for system-level improvements to PMLs. Improvements such as standardised and legible bilingual medication instructions and/or pictograms would appreciably facilitate medication counselling and allow for better understanding of PMLs by older Singaporeans.

